# Liberal Utilitarianism—Yes, But for Whom?

**DOI:** 10.1017/S0963180120000894

**Published:** 2020-10-09

**Authors:** JOONA RÄSÄNEN

## Abstract

In his important paper “Just Better Utilitarianism,” Matti Häyry reminds his readers that liberal utilitarianism can offer a basis for moral and political choices in bioethics and thus could be helpful in decisionmaking.^1^ Although I agree with the general defense of Häyry’s liberal utilitarianism, in this commentary, I urge Häyry to say more on who belongs to our moral community. I challenge Häyry’s principle of actual or prospective existence. I also argue that Häyry should say more on human beings at the “margin of life” (such as fetuses and other mindless humans). I claim that debate over whether some form of utilitarianism is superior over other moral theories is not as important as answering the question underlying these issues: Who belongs to our moral community?

## Introduction

In his important paper “Just Better Utilitarianism,” Matti Häyry reminds his readers that liberal utilitarianism can offer a basis for moral and political choices in bioethics and thus could be helpful in decisionmaking.^1^ Although I agree with the general defense of Häyry’s liberal utilitarianism, in this commentary, I urge Häyry to say more on who belongs to our moral community. I challenge Häyry’s principle of actual or prospective existence. I also argue that Häyry should say more on human beings at the “margin of life” (such as fetuses). I claim that debate over whether some form of utilitarianism is superior over other moral theories is not as important as answering the question underlying these issues: Who belongs to our moral community?

## Challenging the Principle of Actual and Prospective Existence

Häyry’s liberal utilitarianism includes the following principle:

“When the moral rightness of human activities is assessed, the imagined needs of non-existent beings who will never come into existence shall not be counted.”

Call this *the principle of actual or prospective existence.* Häyry adopts this rule to avoid the repugnant conclusion that we must reproduce every time we could have offspring with tolerable lives. This principle is in line with Häyry’s antinatalist view: not having children is both rational and ethical.^2^ ^,^^3^ Some see this sort of antinatalist conclusion as repugnant or implausible itself,^4^ whereas others endorse similar conclusions for somewhat different reasons.^5^ ^,^^6^

I am not sure whether it is wrong to have children. That is because I am not fully confident that existence is always bad. However, I am confident that nonexistence cannot be bad, so it cannot be wrong *not* to have children. Thus, abstaining from procreation seems to be the safe option, morally, because you cannot wrong someone who does not exist. Be that as it may, I think we have a reason to reject the principle of actual or prospective existence or, at least, to revise it.

To see this, consider the following case:

A couple wants to have a child. If they procreate now, their child will be sick. She will suffer pain and discomfort through her life. However, if the couple waits a month, they will have a healthy child whose life is much better – overall – than the life of the child who would be conceived earlier.^7^

Assuming that the child would be a different child because of different DNA and that the couple has no reason not to wait a month, it seems that they *should* wait a month. It is better, morally, to have a child whose life is better than one whose life is worse, other things being equal. Based on some of Häyry’s previous work, I assume he agrees.^8^

However, if the principle of actual or prospective existence is correct, it might be difficult to claim that the couple should wait and have the child whose life would be better instead of proceeding immediately to have the child whose life would be worse. After all, if they choose not to wait a month and have the sick child instead, the other child would never come into existence. If the other child never comes into existence, then according to the principle, her imagined needs are not to be counted. And if her imagined needs are not counted, it is not obvious why the couple should have waited a month and created the better off-child rather than the worse-off child.

So, to avoid this problem, it could be that the imagined needs of people that never come into existence matter, at least sometimes. More precisely, they matter when one has decided to bring a person into existence.

One might wonder what sort of moral obligations the couple have if they cannot have the healthier child at all. For example, suppose that no matter what they do, any child they have will spend her life in pain. Technically, a child they conceived at a later time would be a different person from one they conceived at an earlier time, because postponing the act of procreation would cause different gametes to unite. Would it be wrong for the couple to procreate?

I think many people would agree that if the life of the child is worth living, the couple does nothing morally wrong in bringing her into existence. And many would say that even if they also agreed that a couple that *could* bring a healthy child into existence but intentionally chooses to have a sick child instead does do something wrong.

As I see it, Häyry has three options here. He could reject the principle of actual or prospective existence. But that would, it seems, lead to the repugnant conclusion that we should reproduce every time we could have offspring with tolerable lives. Another choice is to simply bite the bullet and accept that it is not morally wrong to create a life that is worse than some other life you could create instead. But this would contradict Häyry’s previous claims.^9^ The third option, which I think is the most plausible one, is to revise the principle of actual and prospective existence so that it is not vulnerable to the counter-example raised above.

Here is one friendly suggestion for how to do that, which I call *the revised principle of actual or prospective existence*:

When the moral rightness of human activities is assessed, the imagined needs of nonexistent beings who will never come into existence shall not be counted unless one has already made the decision to bring a person into existence. If one has decided to procreate, the imagined needs of nonexistent beings should be counted and one therefore has a moral reason to bring the best-off person one can into existence.

So, if the quality of life of those people who never exist does not matter when one has not decided whether to bring any persons into existence, but only when one has decided to bring a person into existence, the principle does not create an obligation to procreate every time one could do so. This would be in line with what Häyry and others have argued or assumed to be true.^10^

## Do Actual but Mindless Humans Deserve our Moral Consideration?

How we treat mindless humans could also pose a problem for liberal utilitarianism. By *mindless humans*, I mean beings that are biologically human (that have human DNA) but that are not conscious, such as (at least early) fetuses and brain-dead humans. For simplicity, here my discussion is focused on fetuses.

Häyry does not discuss the ethics of abortion or the moral status of the fetuses in his paper, but he mentions Alberto Giubilini and Francesca Minerva’s now-famous article on the moral permissibility of infanticide.^11^ Häyry approaches that article at a more abstract level: his reaction to it was to demand clarity in bioethical arguments^12^ and to discuss the possibility of anonymous publishing.^13^

I assume Häyry’s position on ethics of abortion has not changed significantly since he started his career in philosophical bioethics. Then, Häyry summarized his view as follows: abortion is morally permissible and should be legally permitted as long as the woman makes the decision while being aware of the consequences of her decision to herself and the fetus.^14^

Pro-choice views on the ethics of abortion can, roughly, be based on two kind of arguments: (1) person-denying arguments and (2) bodily-autonomy arguments. Thus, if abortion is not morally wrong, that is so because either (1) a fetus is not a person and does not have a right to life, which means that a fetus is a sort of being whose life is not wrong to end or (2) the pregnant woman has a right to her bodily autonomy, which means that, even if the fetus *has* a right to life or *is* a person, the fetus does not have a right to use another person’s body to sustain its life and, therefore, abortion is morally justified.

In “Just Better Utilitarianism,” Häyry posits his liberal utilitarianism in light of Jeremy Bentham’s words: “The question is not, Can they *reason*? nor, Can they *talk*? but, Can they *suffer*?”^15^ Häyry uses this reasoning on nonhuman animals. According to liberal utilitarianism, meat consumption, factory farming, and other related practices are immoral because animals can, indeed, suffer.^16^ Their interest in not suffering is more basic than our need (or desire, to say it more accurately) to consume animal-based products, such as meat. Because it is wrong to satisfy less basic needs of one being by preventing the possibility of satisfying more basic needs of others, meat consumption and the other practices are morally wrong.

Now, if mindless humans such as fetuses can suffer (i.e., feel pain), could that undermine the notion that liberal utilitarianism justifies abortion? One might think so, because if abortion is permissible, then the more basic needs of the fetuses (avoiding suffering) would be ignored in favor of the less basic needs of pregnant women (controlling what happens to one’s body).

Studies often suggest that a cortex and intact thalamocortical tracts are necessary to experience pain. Since the cortex only becomes functional and the tracts only develop after 24 weeks, many studies hold that a fetus cannot experience pain until the final trimester. But in recent work, it has been argued that neuroscience cannot definitively rule out fetal pain before 24 weeks.^17^ Although most abortions occur well before 24 weeks, some of the States in United States allow abortion even during the final trimester.^18^

Suppose fetuses do feel pain. It seems that liberal utilitarians should at least be concerned about it. They could not simply ignore fetal pain.

In a landmark paper, Judith Jarvis Thomson argued that abortion is still morally permissible even on the assumption that fetuses have a right to life and are persons. To support her position, she offered the following hypothetical case.

***Famous Violinist**.* You wake up and find yourself in a bed, attached to an unconscious famous violinist. He has been found to have a fatal kidney ailment, and the Society of Music Lovers has attached him to you because you alone have the right blood type to help. A doctor tells you: “We’re sorry you have been connected to this person. We would have never allowed it if we had known. But to unplug him now would kill him. Nevertheless, it’s only for nine months; after that, he will recover from his ailment and can safely be unplugged from you.”^19^

Thomson’s reaction to the case was that although it would be very nice for you to remain attached, it is not your moral obligation. It is morally permissible for you to detach yourself from the violinist because, although he has a right to life, he does not have a right to use your body to sustain his own life. Because the case is, allegedly, analogous enough with pregnancy, the pregnant woman likewise has a right to detach the fetus from her, even in the cases where the fetus would die as a result.^20^

To see what moral weight fetal pain, if it exists, would have, we can revise Thomson’s case so that detaching yourself from the violinist is very painful to him. Consider the following revised case.

***Painful Detachment.*** You wake up and find yourself in a bed attached to an unconscious famous violinist. He has been found to have a fatal kidney ailment, and the Society of Music Lovers has attached him to you because you alone have the right blood type to help. A doctor tells you: “We’re sorry you have been connected to this person. We would never have allowed it if we had known. But to unplug him now would kill him *very painfully.* Nevertheless, it is only for nine months; after that, he will cover from his ailment and can safely be unplugged from you.”

Does the pain detaching yourself would cause make you morally obligated to remain attached to the violinist? Probably not. Does it give you a moral reason to consider whether you can do something to ease the pain? Probably yes. If it is possible, the violinist should be offered pain relief, if pain, as a liberal utilitarian must assume, has any moral relevance. Similarly, we should be concerned about fetal pain.

But now, what if we could ease the pain of animals in factory farming? If, given that fetuses do feel pain, we are only obligated to ensure that abortion does not cause the fetus to feel pain, not to prevent abortions per se, why we should stop killing animals? Is not enough that we make sure they are killed painlessly? If on the other hand, we believe that we should not kill animals for food even if we can do so without inflicting pain, why we should not abstain from killing fetuses as well?

A liberal utilitarian might have at least two replies. She might say that *killing animals is unnecessary* because the same goods can be achieved by other means, such as by eating plant-based food. However, there are no proper alternatives to abortion: the same goods that are achieved by abortion cannot be achieved by, for example, gestating the fetus to the term and giving it up for adoption.^21^ One might also say that pregnancies happen inside women’s bodies while killing animals does not, and that this is morally relevant.

But now suppose we imagine that, thanks to new technology, it is no longer necessary to kill the fetus when ending the pregnancy prematurely. Perhaps sometimes, pregnancy does *not* happen inside a female body in the first place.^22^ Consider the following cases.

***Partial ectogenesis.*** It has become possible to detach a fetus from the female body in a very early phase of pregnancy and gestate the fetus inside an artificial womb instead. A woman gets pregnant and wants to have an abortion, but the doctor tells her: “You know, you don't need to kill the fetus. We can just remove it alive and gestate it in an artificial womb machine. Then, after a few months, you could give it up for adoption if you still feel you don’t want to have a child.”

Is the woman still entitled to have the fetus killed, or is she morally obligated *not* to kill the fetus? Surely, people’s intuitions differ here, but I suspect—and some studies suggest^23^—that at least many women would feel that avoiding the burdens of the pregnancy is not the point of abortion: the point of abortion is not to have a child at all.^24^ ^,^^25^ ^,^^26^

To propose partial ectogenesis as an alternative to abortion would thus be to misunderstand the purpose of abortion. Since the *purpose* of the abortion is to have the fetus killed, but the *justification* for the abortion is bodily autonomy and integrity, when an artificial womb device becomes an option, another justification for abortion will be needed. That brings us to the next case.

***Complete ectogenesis.*** It has become possible to create embryos in vitro and gestate them in artificial womb machines. A couple wants to have a child, but after the embryos are created and transferred into the machine, they change their mind. They do not want to have a child. However, the doctor tells them: “You know, you don’t need to destroy the embryo developing in the machine. You can just leave it there, and when the time comes, if you do not want the newborn, we can give it to some other couple that does want to have a child.”

Is the couple—either together or separately—still entitled to have the embryo destroyed, or are they morally obligated *not* to destroy it?^27^ ^,^^28^ ^,^^29^ ^,^^30^ Again, our intuitions probably differ. But what seems to be relevant is whether the fetus itself is the sort of being whose life it is seriously morally wrong to end. So, it is likely that to determine what the couple or the woman should do, morally, in the above cases cannot be answered without answering the question of whether the fetus itself is entitled to a right to life. Häyry could just assume that embryos or fetuses are not the sort of beings (persons) whose life it is seriously morally wrong to end. But this simply assumes an answer to the very difficult question that, to my mind, should be answered first.

It is very easy to be a utilitarian when faced with simple scenarios. It is also easy to become a utilitarian in time of crisis: for instance, public health often uses a utilitarian approach to make triage decisions during pandemics^31^ such as COVID-19. For an example of a simple case, consider the following.

A billionaire who is convinced by liberal utilitarianism donates one million dollars to the state on the condition that the money be spent to save as many of his fellow citizens’ lives as possible. The state has two choices: use the money for one ambulance helicopter that patrols a rural part of the country, or use the money for 10 ambulances that are used in major cities. The helicopter would save, on average, one life annually while the ambulances would save, on average, one hundred lives annually.

It is obvious what to choose. Other things being equal, the money should be spent for the ambulances, so that the greatest number of people would be saved.

But now suppose there is a third possibility. Use the money to fund a campaign to discourage women from choosing abortion. Suppose further that this money would save 1,000 embryos and fetuses from being aborted. Should the state choose this policy instead because it saves even more lives? It depends. It depends on whether we count fetuses and embryos as part of our moral community. If we do, then it seems to be a moral obligation to choose the campaign, but if we do not consider these mindless humans a part of our moral community, then there is no moral obligation to choose this option over the ambulances.^32^

There are also recent real-life cases that illustrate the problem. For instance, there is an ongoing debate whether guidelines for treating extremely premature babies should be altered to free up ventilators for adults during the COVID-19 pandemic.^33^ In some cases, a ventilator would give an adult a higher probability of survival than it would give the extremely premature baby who would otherwise get it. However, saving babies rather than adults would likely maximize life years saved, since a baby who survives is likely to live longer than an adult who does. It is difficult to apply any utilitarian approach successfully if we do not know what moral status to assign to the mindless human fetuses.^34^

We could simply say that fetuses are not persons because they lack (self-)consciousness. But we could say many nonhuman animals lack that as well. Or someone could reply that being a person does not matter: what matters is that when killing a fetus (or embryo) we are depriving it of life unjustly.^35^ Is not life itself a very basic need that outweighs any alleged needs or wants to control one’s body?

It is very easy to be a utilitarian when it is clear who belongs to our moral community. But it is much more difficult to apply utilitarian approaches to practical issues when there is a reasonable disagreement as to whether someone (or something) is a sort of being we should be morally concerned about. Consider (illegal) immigrants, recipients of international aid, fetuses and embryos, the brain-dead, the severely mentally disabled, animals, and so on.^36^

How societies should treat the aforementioned is not obvious because it is not obvious whether they belong to our moral community. I am afraid that liberal utilitarianism cannot tell us how we should treat them unless we somehow determine whether they are the sort of beings we should be morally concerned about.

## Notes

1. Häyry M. Just better utilitarianism. *Cambridge Quarterly of Healthcare Ethics* 2021;30(2). https://doi.org/10.1017/S0963180120000882. See also Häyry M. Liberal Utilitarianism and Applied Ethics. London, UK: Routledge; 1994.

2.

Häyry M. A rational cure for prereproductive stress syndrome. Journal of Medical Ethics 2004;30:377–78.1528952510.1136/jme.2003.004424PMC1733883

3.

Häyry M. The rational cure for prereproductive stress syndrome revisited. Journal of Medical Ethics 2005;31:606–7.1619960510.1136/jme.2005.011684PMC1734020

4.

Pihlström S. Ethical unthinkabilities and philosophical seriousness. Metaphilosophy 2009;40:656–70.

5.

Benatar D. Better *Never to Have Been: The Harm of Coming Into Existence*. Oxford: Oxford University Press; 2008.

6.

Rachels S. The immorality of having children. Ethical Theory and Moral Practice 2014;17:567–82.

7. Derek Parfit famously raised this kind of example in Parfit D. Reasons and Persons. Oxford: Oxford University Press; 1984.

8.

Häyry M. If you must make babies, then at least make the best babies you can? Human Fertility 2004;7:105–12.1522375910.1080/14647270410001699063

9. Häyry could also say that there has been philosophical progress in his view and that he no longer thinks one should create the best possible lives if one is going to procreate. Or he could claim that he does not actually believe (some of) his arguments. At least one scholar has claimed that a philosopher does not have to believe himself what he or she is arguing for. See Plakias, A. Publishing without belief. Analysis 2019;79:638–46.

10.

Savulescu J. Procreative beneficence: Why we should select the best children. Bioethics 2008;15:413–2610.1111/1467-8519.0025112058767. Also, for example, Peter Singer says: “To focus only on those who exist or will exist anyway leaves out something vital to the ethics of this decision [which lives to create]. We can, and we should, compare the lives of those who will exist with the lives of those who might have existed, if we had acted differently. …We can and should ‘argue as if from the abyss of the nonexistent.’ Never having tasted ‘life’s desire,’ they will ‘feel no dearth’ of life. Yet the quality of the lives they would have led is inescapably relevant to our decision.” Singer P. Practical Ethics. 3rd ed. Cambridge: Cambridge University Press; 2011, at 110–1.

11.

Giubilini A , Minerva F. After-birth abortion: why should the baby live? Journal of Medical Ethics 2013;39:261–32236129610.1136/medethics-2011-100411. For other arguments that infanticide is (in some cases) morally permissible, see Kuhse H , Singer P. Should the Baby Live? The Problem of Handicapped Infants. Oxford: Oxford University Press; 1983 ; Hassoun N , Kriegel U. Consciousness and the moral permissibility of infanticide. Journal of Applied Philosophy 2008;25:45–55 ; Räsänen J. Pro-life arguments against infanticide and why they are not convincing. Bioethics 2016;30:656–62.2771705810.1111/bioe.12281

12.

Häyry M. What exactly did you claim? A call for clarity in the presentation of premises and conclusions in philosophical contributions to ethics. Cambridge Quarterly of Healthcare Ethics 2015;24:107–12.2547386310.1017/S0963180114000358

13.

Häyry M. Academic freedom, public reactions, and anonymity. Bioethics 2014;28:170–3.2472454110.1111/bioe.12094

14.

Häyry M , Häyry H. Rakasta, *Kärsi ja Unhoita: Moraalifilosofisia Pohdintoja Ihmiselämän Alusta ja Lopusta*. [Love, Suffer and Forget: Moral philosophical thoughts on the beginning and end of human life, in Finnish] Helsinki: Kirjayhtymä; 1987.

15.

Bentham J. An Introduction to the Principles of Morals and Legislation. Burns JH , Hart HLA , eds. London and New York: Methuen; 1982 [1789], at 283 as cited in Häyry 2020, note 1.

16. A famous utilitarian argument for animal rights was made in Singer P. Animal Liberation. New York, NY: Harper Collins; 1975.

17.

Derbyshire S , Bockmann J. Reconsidering fetal pain. Journal of Medical Ethics 2020;46:3–6.3193766910.1136/medethics-2019-105701

18. For instance, while the landmark decision in the United States (*Roe v. Wade*) prohibited states from banning abortion in the first trimester, the states are free to allow abortion during the second and third trimesters as well.

19. Adapted from Thomson J. J. A defense of abortion. Philosophy & Public Affairs 1971;1:–66.

20. There are disagreements on whether her argument manages to justify abortion. For a recent criticism, see for example Bernstein C , Manata P. Moral responsibility and the wrongness of abortion. Journal of Medicine and Philosophy 2019;44:243–62.3087777710.1093/jmp/jhy039

21.

Räsänen J. Against the impairment principle: A reply to Hendricks. Bioethics 2020; 34:862–64.3201715810.1111/bioe.12720

22.

Räsänen J , Smajdor A. The ethics of ectogenesis. Bioethics 2020;34:328–30.3233368810.1111/bioe.12745

23.

Cannold L. Women, ectogenesis, and ethical theory. Journal of Applied Philosophy 1995;12:55–64.1241652410.1111/j.1468-5930.1995.tb00119.x

24.

Langford S. An end to abortion? A feminist critique of the ‘ectogenetic solution’ to abortion’. Women's Studies International Forum 2008;31:–9.

25.

Mackenzie C. Abortion and embodiment. Australasian Journal of Philosophy 1992;70:136–55.1165959610.1080/00048409212345041

26.

Overall C. Rethinking abortion, ectogenesis, and fetal death. Journal of Social Philosophy 2015;46:126–40.

27.

Räsänen J. Ectogenesis, abortion and a right to the death of the fetus. Bioethics 2017;31:697–702.2904469510.1111/bioe.12404

28.

Hendricks P. There is no right to the death of the fetus. Bioethics 2018;32:395–7.2988351910.1111/bioe.12455

29.

Kaczor C. Ectogenesis and a right to the death of the prenatal human being: A reply to Räsänen. Bioethics 2018;32:634–8.3025294410.1111/bioe.12512

30.

Blackshaw B , Rodger D. Ectogenesis and the case against the right to the death of the foetus. Bioethics 2019;33:76–81.3034192310.1111/bioe.12529

31.

Childress J , Faden R , Gaare R , Gostin L , Kahn J , Bonnie R , et al. Public health ethics: Mapping the terrain. Journal of Law, Medicine and Ethics 2002;30:170–8.10.1111/j.1748-720x.2002.tb00384.x12066595

32. In abortion ethics literature, there is a vast amount of work on a similar thought experiment (embryo rescue case) that aims to show that embryos are not persons. See Liao M. The embryo rescue case. Theoretical Medicine and Bioethics 2006;27:141–710.1007/s11017-005-1390-z16699964; Räsänen J. Why pro‐life arguments still are not convincing: A reply to my critics. Bioethics 2018;32:628–333016886310.1111/bioe.12502; Hendricks P. Why the embryo rescue case is a bad argument against embryonic personhood. Bioethics 2019;33:669–733110755710.1111/bioe.12588; Hershenov D. What must pro-lifers believe about the moral status of embryos? Pacific Philospohical Quarterly. 2020;101:186–202.

33.

Haward M , Janvier A , Moore G , Fry J , Laventhal N , Lantos J. Should extremely premature babies get ventilators during the COVID-19 crisis? American Journal of Bioethics 2020;20:37–43.10.1080/15265161.2020.176413432400291

34.

Räsänen, J. Saving the babies or the elderly in a time of crisis? American Journal of Bioethics 2020;20:180–2.3271677310.1080/15265161.2020.1779866

35.

Marquis D. Why abortion is immoral. Journal of Philosophy 1989;86:183–202.11782094

36. Häyry briefly discusses some of the mentioned entities in his *Just Better Utilitarianism* and in Häyry M. Causation, responsibility, and harm: How the discursive shift from law and ethics to social justice sealed the plight of nonhuman animals. Cambridge Quarterly of Healthcare Ethics 2020;29:246–67.3215948810.1017/S096318011900104X

